# Unicornuate Uterus With a Rudimentary Horn Adenomyoma, Ipsilateral Renal Agenesis, and Deep Infiltrating Endometriosis: A Case Report

**DOI:** 10.7759/cureus.116310

**Published:** 2026-09-15

**Authors:** Tiago Romão, Miriam Marques, José Reis, Adalgisa Guerra

**Affiliations:** 1 Radiology, Hospital Beatriz Ângelo, Loures, PRT; 2 Gynecology, Hospital da Luz, Lisbon, PRT; 3 Radiology, Hospital da Luz, Lisbon, PRT

**Keywords:** adenomyoma, deep infiltrating endometriosis, müllerian duct anomaly, pelvic mri, renal agenesis, rudimentary horn, unicornuate uterus

## Abstract

Congenital uterine malformations are often difficult to classify, and an incorrect label can carry over for years across successive clinical records. We describe a 39-year-old woman, gravida 1 para 1, referred for chronic pelvic pain and intermenstrual bleeding, whose uterus had previously been recorded as didelphys. Pelvic magnetic resonance imaging, with T2-weighted sequences in three planes, T1-weighted and fat-saturated sequences, diffusion-weighted imaging, and post-gadolinium imaging, instead demonstrated a unicornuate uterus with a left, cavitated, non-communicating functional rudimentary horn (European Society of Human Reproduction and Embryology/European Society for Gynaecological Endoscopy (ESHRE/ESGE) class U4a; American Society for Reproductive Medicine (ASRM) unicornuate uterus with a non-communicating rudimentary horn). The rudimentary horn was almost entirely occupied by a 6 cm adenomyoma, with a small residual endometrial-lined cavity; the contralateral functional horn showed diffuse adenomyosis; a deep infiltrating endometriotic nodule extended to the left pelvic sidewall with neurovascular tethering and rectal tenting but no transmural invasion; the left ovary contained an endometrioma; and the left kidney was absent. Surgery and histopathology confirmed a single cervix with a rudimentary horn fused at the cervical level, together with the adenomyoma, extensive adenomyosis, and ovarian endometriosis; the patient recovered without complications and became asymptomatic. The case shows how imaging settled a long-standing classification error and how a single examination characterized the associated renal and endometriotic findings that follow from the shared embryology of the genital and urinary tracts.

## Introduction

Congenital uterine anomalies result from abnormal development of the Müllerian ducts; they affect an estimated 4%-7% of women and are associated with health and reproductive problems, including effects on menstruation, fertility, pregnancy, and, through shared embryology, the urinary tract [1,2]. A unicornuate uterus develops when only one Müllerian duct forms normally and the other arrests, leaving a single developed uterine cavity. In many of these cases, an underdeveloped second uterine structure, termed a rudimentary horn, persists; its clinical weight depends on whether it contains functional (hormone-responsive) endometrium and whether this cavity communicates with the unicornuate cavity [1,2]. When a functional horn does not communicate, menstrual blood can accumulate within it - a condition called hematometra - which can also drive retrograde menstruation (backward flow of menstrual blood through the fallopian tubes) and endometriosis, and rarely allows an ectopic pregnancy within the horn that carries a high risk of rupture [3]. The mesonephric duct guides the developing Müllerian duct and also gives rise to the ureteric bud; a developmental insult on one side therefore commonly affects both structures, leaving a genital anomaly alongside an ipsilateral renal anomaly, most often renal agenesis [4].

The practical difficulty these patients pose is distinguishing a unicornuate uterus with a rudimentary horn from a uterus didelphys: the unicornuate uterus reflects development of only one side, with a single cervix and, when present, an accessory rudimentary horn, whereas uterus didelphys reflects two separately developed uterine halves, each with its own cervix. The two are easily confused on clinical examination and on ultrasound; the distinction instead rests on the number of cervices and on how any accessory horn relates to the cervix, features that cross-sectional imaging is well suited to resolve [5]. We report a woman whose malformation had been labeled didelphys for years and was reclassified on magnetic resonance imaging (MRI) as a unicornuate uterus with a rudimentary horn harboring an adenomyoma, with ipsilateral renal agenesis and severe endometriosis - a combination that MRI resolved in a single examination.

## Case presentation

History

A 39-year-old woman, gravida 1 para 1, with one delivery at 37 weeks complicated by pre-eclampsia seven years earlier, presented for reassessment of chronic cyclical pelvic pain, intermittent intermenstrual spotting, and occasional dyschezia. She had no dyspareunia, urinary symptoms, or fever. She carried long-standing diagnoses of a complex uterine malformation, absence of the left kidney, and endometriosis. Combined hormonal contraception had been stopped three months earlier; at reassessment, her pelvic pain had eased slightly, but her other symptoms persisted. Her surgical history included an ectopic umbilicus in the hypogastrium with an umbilical hernia, corrected at a previous operation. An earlier transvaginal ultrasound had shown a bulky left adnexal mass of about 70 × 67 mm (two orthogonal diameters), with the left ovary not separately seen; the study characterized the mass as of uncertain origin - a possible adenomyoma, leiomyoma, or pelvic kidney - and did not clarify the uterine anatomy or the number of cervices.

Examination

Speculum examination showed a healthy cervix; the patient was menstruating at the time. Bimanual palpation revealed no rectovaginal septum nodularity, a mobile uterus, and a mobile 6 cm anterior uterine nodule.

Imaging

Pelvic MRI was performed on a 1.5 T system, without intravaginal gel. The protocol comprised T2-weighted sequences in the sagittal, axial, and coronal planes, T1-weighted and T1-weighted fat-saturated sequences, diffusion-weighted imaging, and T1-weighted imaging after gadolinium administration. A single functional horn on the right carried a cervix and showed diffuse junctional-zone thickening in keeping with adenomyosis. Connected to the left aspect of the cervix lay a rudimentary left horn, almost entirely occupied by a nodular mass with signal features favoring an adenomyoma (subsequently confirmed at histopathology), 6 cm in its largest axis - corresponding to the same lesion seen on the earlier ultrasound, the apparent difference in size likely reflecting differences in measurement plane and the difficulty of defining its margins on ultrasound; a small residual endometrial-lined cavity within this adenomyomatous horn contained an associated apparent hematometra measuring about 8 mm in maximum diameter, with no associated dilation of the functional right-sided cavity, a pattern consistent with a non-communicating configuration. Hysterosalpingography, hysteroscopy, and intraoperative probing were not performed to confirm patency directly. A fibrotic deep infiltrating endometriotic nodule abutted the right cervix and the inferior margin of the adenomyoma and extended to the left pelvic sidewall, tethering the neurovascular bundle; on midline sagittal T2-weighted imaging, it lay 9.5 cm from the anal margin, and it tented the rectum without transmural invasion. The right adnexum sat retro-uterine with a surface hemorrhagic implant; the left adnexum was high, with hemorrhagic cystic content of about 3.3 cm consistent with an endometrioma. There was no uretero-hydronephrosis, and the right ureter ran normally. The left kidney was absent. The bladder wall was not thickened, and there was no pelvic or inguinal lymphadenopathy. The horn mass measured the same dimensions as on a study performed five years earlier, indicating no interval growth (Figures 1-6).

**Figure 1:**
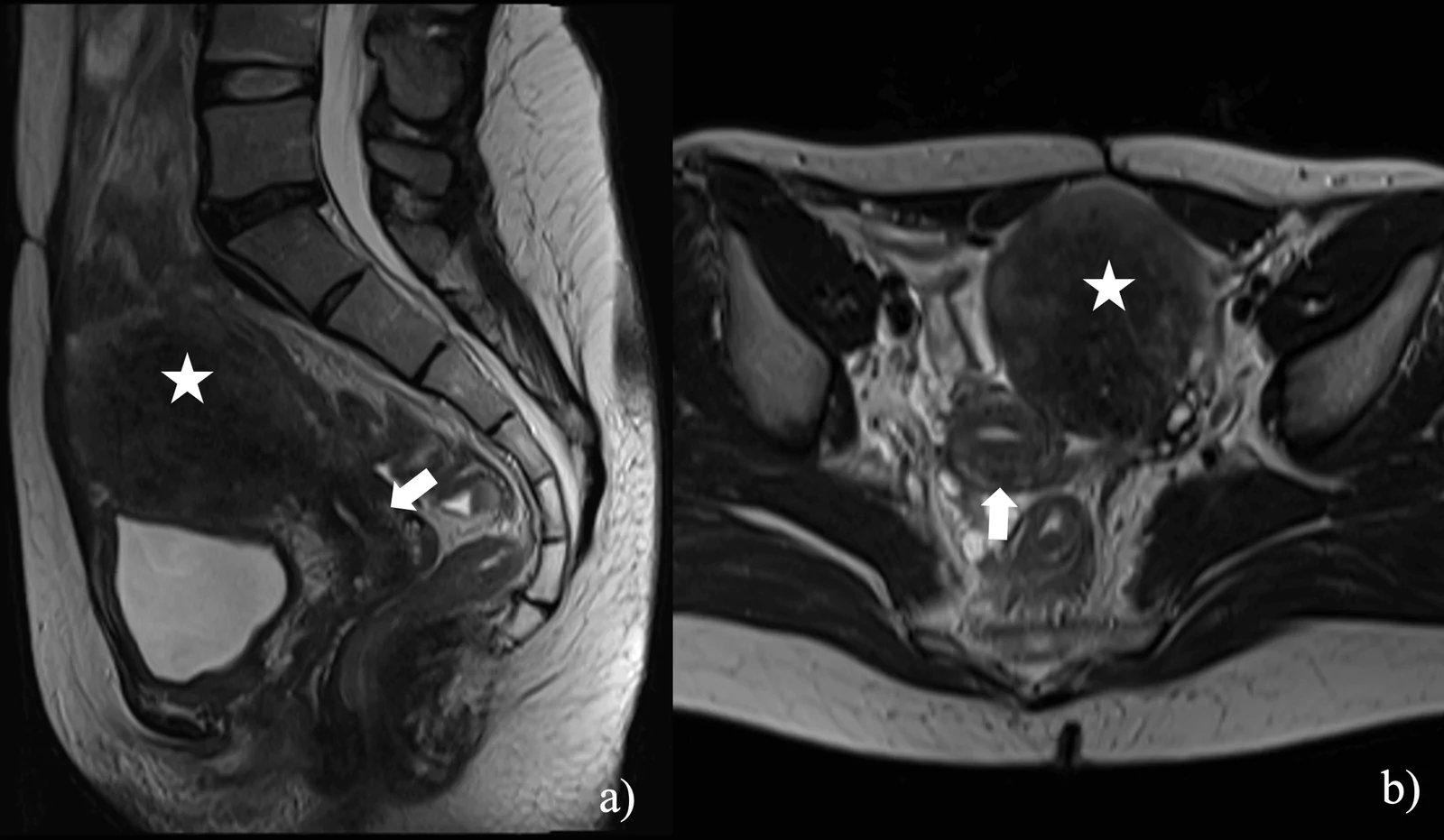
Sagittal (a) and axial (b) T2-weighted magnetic resonance (MR) images in a 39-year-old woman with a Müllerian anomaly show a bulky pelvic mass (star) anterior to the uterine body, with diffuse junctional-zone thickening of the functional horn, consistent with adenomyosis. A single cervix (arrow) is identified, with the mass abutting its left aspect, a finding that argues against uterus didelphys.

**Figure 2:**
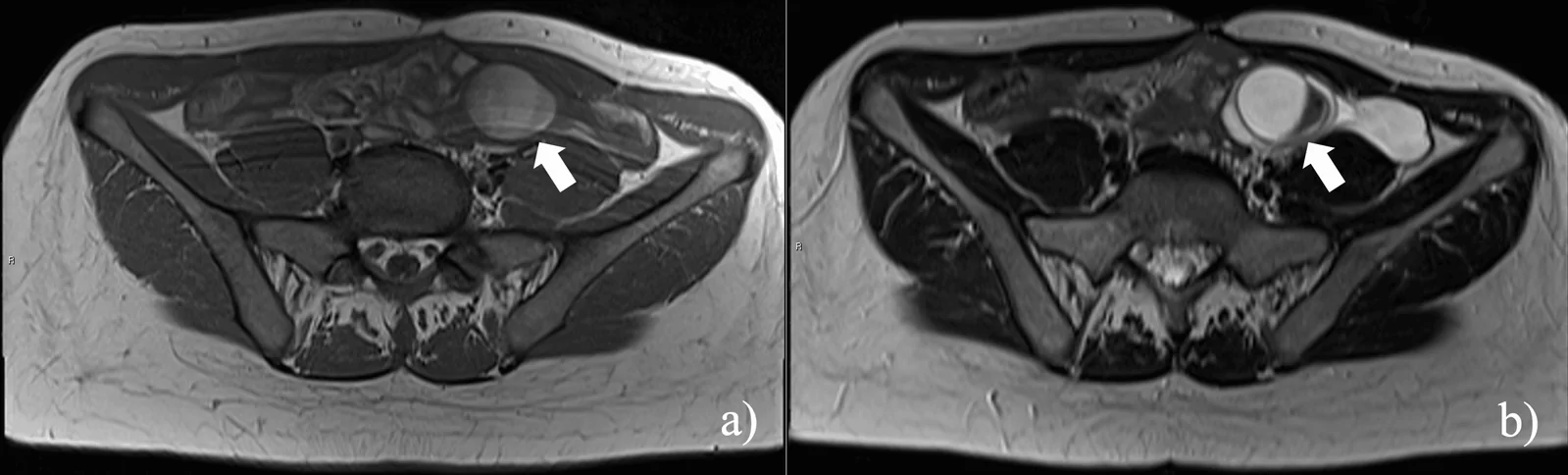
Axial T2-weighted (a) and axial T1-weighted (b) magnetic resonance (MR) images show a high-positioned left adnexal lesion (arrow) with hyperintense cystic content, approximately 3.3 cm in diameter, consistent with a left ovarian endometrioma. The right ovary was unremarkable.

**Figure 3:**
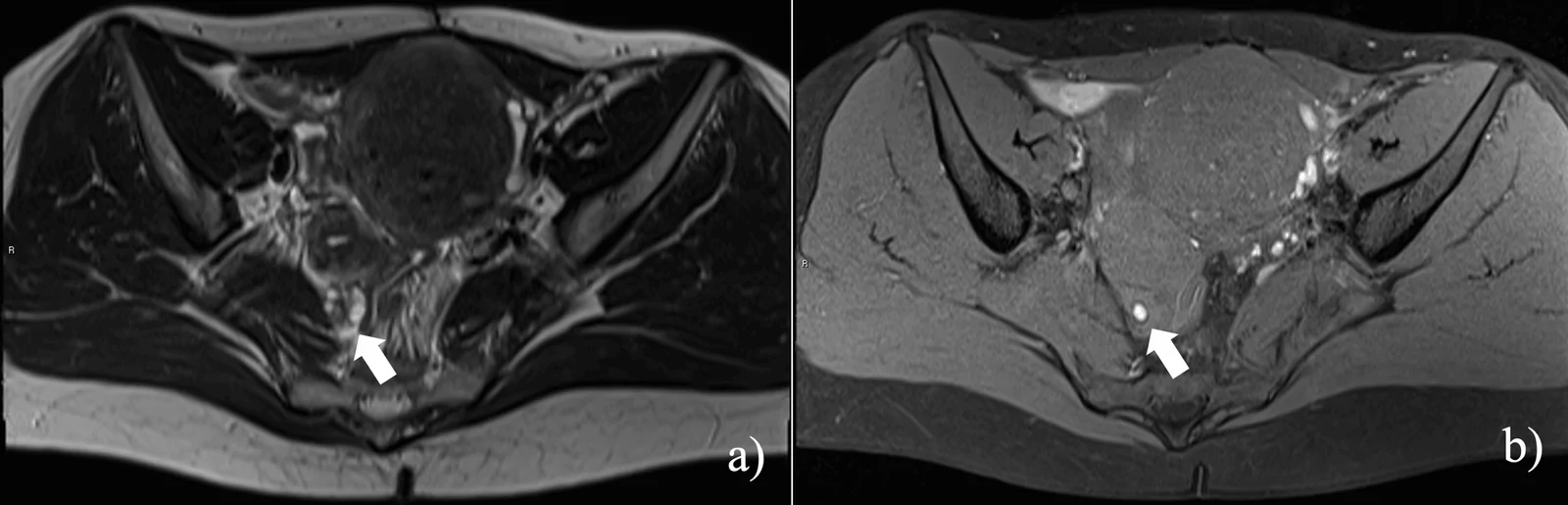
Axial T2-weighted (a) and axial fat-suppressed T1-weighted (b) magnetic resonance (MR) images show a fibrotic deep infiltrating endometriotic nodule of the posterior/left lateral compartment (arrow), extending to the left pelvic sidewall with tethering of the neurovascular bundle. The nodule lay approximately 9.5 cm from the anal margin and tented the rectum without transmural invasion.

**Figure 4:**
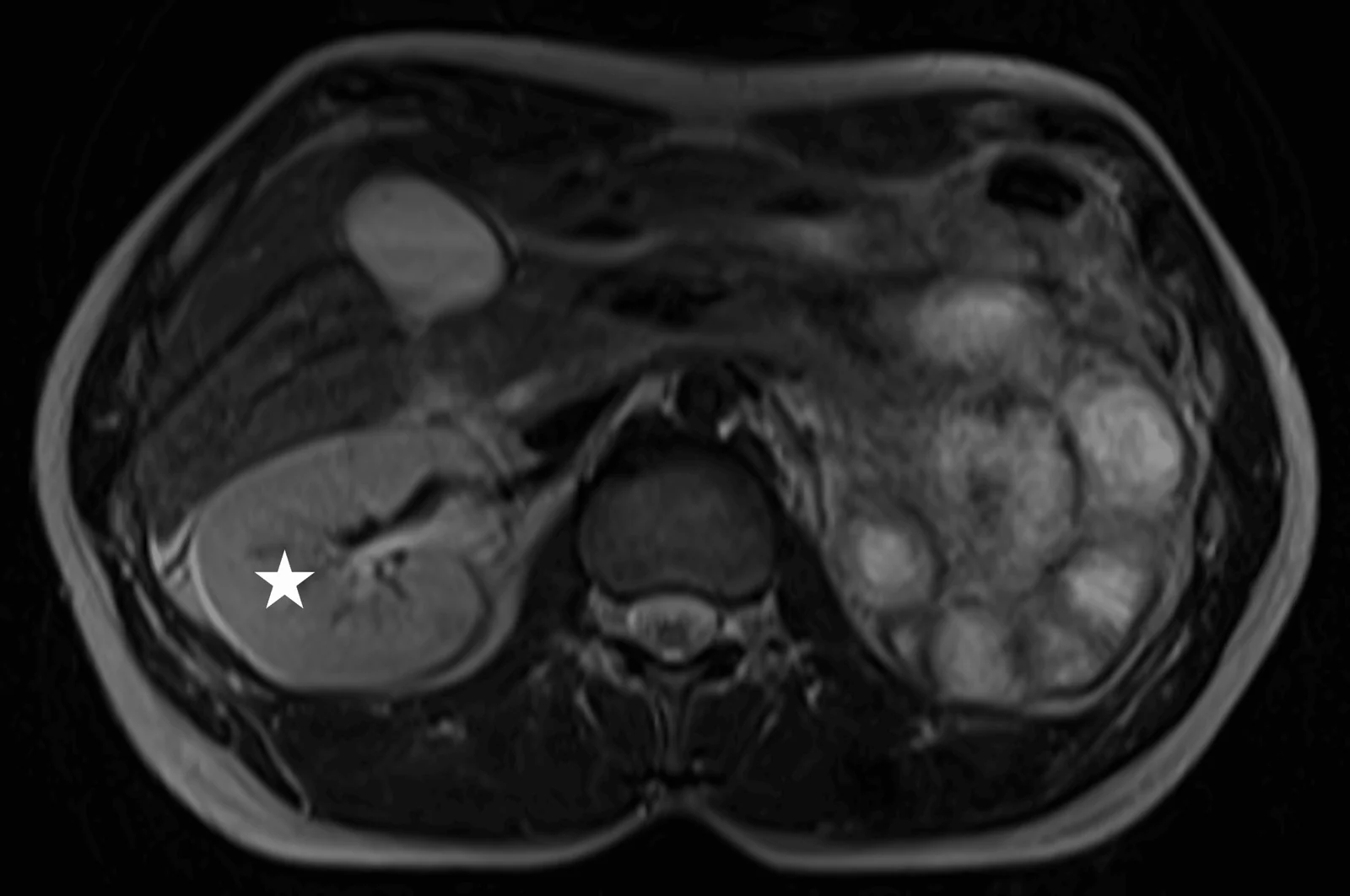
Axial T2-weighted magnetic resonance (MR) image of the abdomen shows a normal right kidney (star) in the right renal fossa. The left renal fossa is occupied by bowel loops, with no left kidney identified, confirming left renal agenesis in association with the ipsilateral Müllerian anomaly.

**Figure 5:**
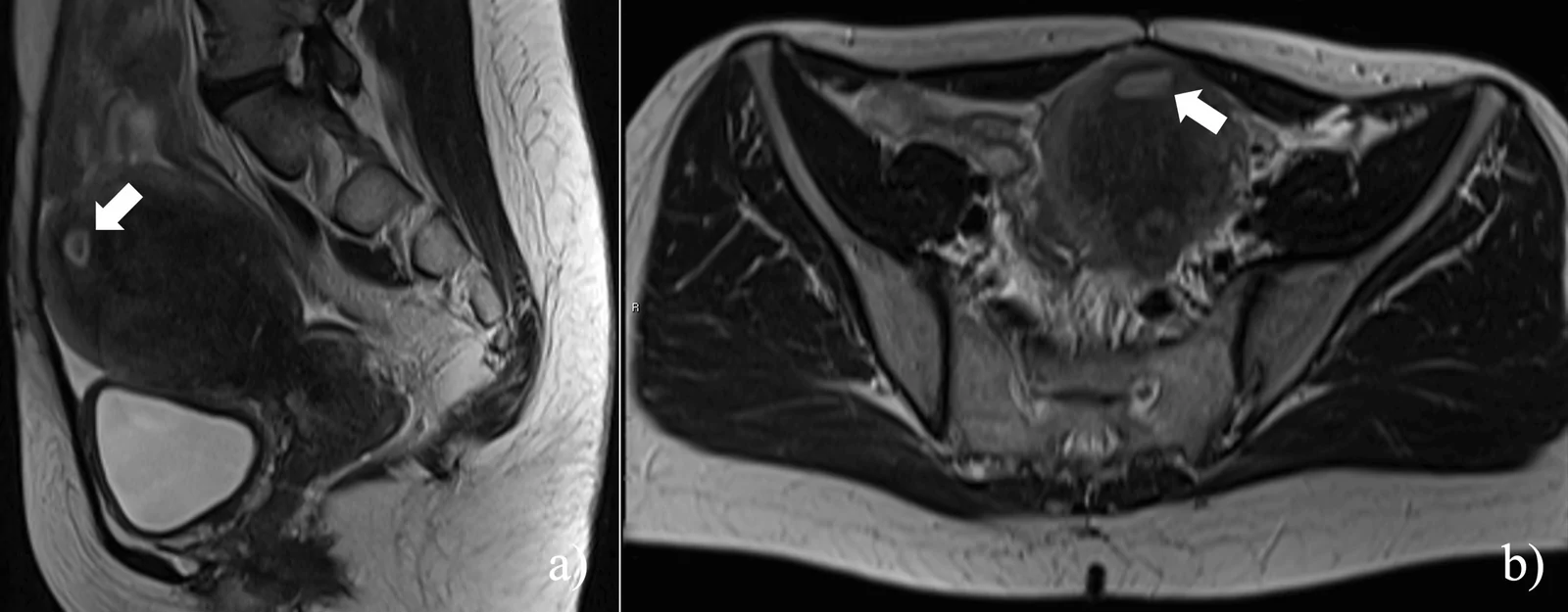
Sagittal (a) and axial (b) T2-weighted magnetic resonance (MR) images show a T2-hyperintense endometrial lining within the left rudimentary horn, with the surrounding junctional zone and myometrium. The functional endometrium supports the diagnosis of a cavitated functional horn rather than a leiomyoma, an adenomyoma without a cavity, or an adnexal mass, and it accounts for the associated hematometra.

**Figure 6:**
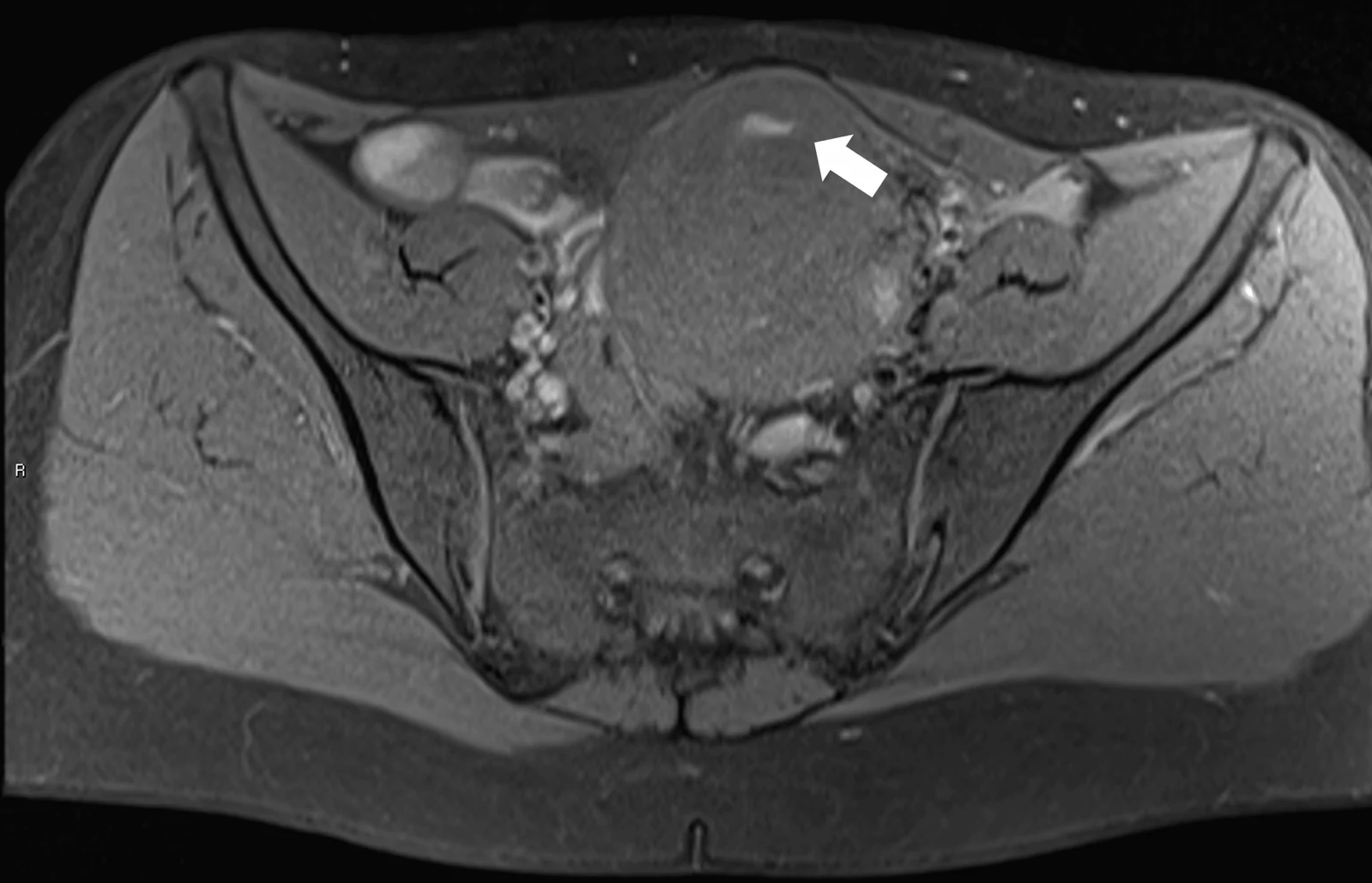
Axial T1-weighted magnetic resonance (MR) image shows a small focus of T1-hyperintense hemorrhagic content within the rudimentary horn cavity, consistent with hematometra.

Management and confirmation

With only slight improvement in pelvic pain after discontinuing oral contraception, persistence of her other symptoms, a completed family, and MRI findings indicating a structural risk unlikely to resolve with hormonal treatment alone (a non-communicating functional horn, extensive adenomyosis, and deep infiltrating endometriosis), the patient chose definitive surgery. Total abdominal hysterectomy was performed by laparotomy, with left adnexectomy, right salpingectomy, and excision of the endometriotic nodule; this showed an enlarged uterus with a single cervix and a left rudimentary horn fused to the uterus at the cervical level, an anatomy that cannot be reconciled with a true didelphys uterus and that matched the imaging diagnosis. The rudimentary horn was almost entirely occupied by the adenomyoma, and a methylene blue test confirmed bladder integrity after a vesical dissection made difficult by the distorted anatomy. Histopathology reported an adenomyoma, extensive and diffuse adenomyosis, and ovarian endometriosis. Recovery was uneventful, and the patient's symptoms resolved. The rudimentary horn and adenomyoma measured 6 cm in its largest axis on MRI; on gross pathology, the excised specimen measured 9.3 × 8.5 × 8.0 cm, weighing 355 g. Serial imaging had excluded interval growth before surgery; the size discrepancy may reflect differences in measurement plane and technique between imaging and gross pathology, and in specimen handling.

## Discussion

This case highlights how an incorrect classification of a Müllerian anomaly may persist for many years and demonstrates the critical role of MRI in achieving accurate anatomical characterization and definitive diagnosis. A didelphys uterus and a unicornuate uterus with a rudimentary horn both present two apparent uterine bodies, which is why ultrasound and clinical examination can confuse them, but they differ where it counts. The didelphys uterus has two complete hemi-uteri, each with its own cervix, and is usually compatible with pregnancy. The unicornuate uterus with a rudimentary horn has a single functional cavity and a single cervix, with an accessory horn that may hold functional endometrium; a non-communicating functional horn predisposes to hematometra [1] and, through retrograde menstruation, to endometriosis [6,7], and rarely to a rudimentary horn pregnancy [3]. In this patient, the single cervix and the cervical-level fusion of the left horn pointed away from didelphys and toward a unicornuate uterus with a rudimentary horn, and surgery bore that out.

On MRI, the decisive observations are the number of cervices and the relationship of the accessory horn to the cervix. The external fundal contour and the relationship between horns are shown best on an oblique coronal T2-weighted sequence aligned to the long axis of the uterus; in this study, the single cervix and the cervical-level fusion of the rudimentary horn were demonstrated across the sagittal, axial, and coronal planes. Aligning the coronal acquisition to the uterine long axis is worthwhile whenever a Müllerian anomaly is suspected, since it most reliably separates the unicornuate, bicornuate, and didelphys patterns [5]. A large adenomyoma within a rudimentary horn can be mistaken for a leiomyoma or an adnexal mass, as the first ultrasound report illustrates; on MRI, it appears as an ill-defined myometrial mass, low on T2, and its poorly defined margins and small cystic foci help to separate it from the sharply marginated leiomyoma, a distinction in which MRI outperforms ultrasound, particularly when leiomyomas coexist [8]. A cystic adenomyoma can itself mimic a uterine anomaly, which is consistent with the diagnostic confusion at the first ultrasound in this patient [8], and post-gadolinium T1 imaging separates the enhancing adenomyoma from the non-enhancing hemorrhagic content of the endometrioma and the hematometra [5]. The reasons ultrasound had failed in this patient are worth stating plainly: its restricted field of view could not encompass a malformation this distorted, the bulky adenomyoma obscured the underlying uterine morphology, the anatomy was displaced by the mass, and transvaginal ultrasound could neither define the cervical anatomy nor establish the number of cervices - the single observation that separates a unicornuate uterus with a rudimentary horn from a didelphys uterus. MRI, with its large field of view, multiplanar capability, and soft-tissue contrast, resolved each of these limitations in one examination [5].

Several entities enter the differential for a solid or partly cystic mass fused to the uterus in this setting, and MRI is what separates them. An accessory cavitated uterine mass (ACUM) is a cavitated lesion of the anterolateral uterine wall lined by functional endometrium, typically at the level of the round ligament insertion in a uterus that is otherwise normally formed [9]; here, the presence of a single functional horn, rather than a normal uterine body, argued against it. A broad ligament (extrauterine) leiomyoma is sharply marginated, low on T2, without an internal cavity or junctional-zone continuity, and does not communicate with the endometrium [10]. A non-communicating functional rudimentary horn is the closest mimic and is in fact the correct diagnosis here, distinguished by its cavity lined by endometrium, the associated hematometra, and its connection to the unicornuate uterus at the level of the cervix. A left adnexal mass - the initial ultrasound interpretation - is excluded by the demonstration of a normal or separately identified ovary and by the lesion's continuity with the myometrium. Given the mass's size, leiomyosarcoma was also considered; established MR features that raise concern for sarcoma - nodular, ill-defined borders, restricted diffusion, T2 dark areas, and size greater than 10 cm [10] - were not present, and the whorled, homogeneous cut surface without necrosis on gross pathology, together with benign histopathology, argued against this diagnosis.

Table 1 summarizes the MRI features that discriminate these possibilities.

**Table 1:** Magnetic resonance imaging (MRI) features distinguishing the main differential diagnoses of a uterine/para-uterine mass in a suspected unilateral Müllerian anomaly. Data collated from references [1,2,5,8-10].

Entity	External contour	Internal cavity/endometrium	Relation to uterus	Key discriminator
Unicornuate uterus with non-communicating functional horn	Single well-formed horn; contralateral rudimentary horn	Functional endometrium in the horn; hematometra	Horn fused to the uterus at cervical level; single cervix	Cavity with endometrium + hematometra; ipsilateral renal agenesis
Uterus didelphys	Two complete, divergent hemi-uteri	Two functional cavities	Two separate cervices	Two cervices (absent here)
Accessory cavitated uterine mass (ACUM)	Normal external contour; single, otherwise normal uterus	Small cavity with functional endometrium	Anterolateral wall, near round ligament; separate from cavity	Normal uterus otherwise; typical location
Broad ligament leiomyoma	Normal uterus	No cavity; no endometrium	Extrauterine; no myometrial continuity	Sharp margins, low T2, no cystic/hemorrhagic cavity
Adnexal mass	Normal uterus	No myometrial cavity	Separate from uterus	Continuity with ovary; myometrium not involved
Adenomyoma	Normal or focally bulging	Tiny cystic foci; junctional-zone continuity	Within/contiguous with myometrium	Ill-defined margins vs. sharply marginated leiomyoma
Leiomyosarcoma	Often nodular, ill-defined	No cavity; may show necrosis/hemorrhage	May invade beyond myometrium	Restricted diffusion, T2 dark areas, size > 10 cm; argued against here by the whorled, homogeneous gross appearance without necrosis and by benign histopathology

The left renal agenesis follows from the same embryology. The mesonephric duct both scaffolds the migrating Müllerian duct and forms the ureteric bud, so a unilateral insult tends to leave a genital anomaly and an ipsilateral renal anomaly together [6]; imaging series confirm the statistical association, with renal agenesis the most frequent renal finding when one occurs [4]. Confirming the presence and position of both kidneys is therefore part of reporting any lateral Müllerian anomaly, and unilateral renal agenesis found incidentally should prompt a look at the uterus. The obstructed hemivagina with ipsilateral renal agenesis syndrome is the classic obstructive version of this association; the present case shares the genital-renal link but has no obstructed hemivagina, a reminder that the embryological association is wider than any single named syndrome [6]. Reaching the correct label matters beyond planning this operation. A unicornuate uterus with a functional rudimentary horn carries a defined set of downstream risks that a diagnosis of didelphys would not have flagged: a rudimentary horn pregnancy with a high risk of rupture and - through the mechanisms already noted - hematometra and endometriosis. The ipsilateral renal anomaly must also be sought [4]. Even where surgery is not immediately planned, the classification therefore shapes counseling, surveillance, and the anticipation of complications.

The association between Müllerian anomalies and endometriosis is well documented and is strongest where a functional segment drains poorly. In obstructive anomalies such as the obstructed hemivagina with ipsilateral renal agenesis, endometriosis is reported in roughly 17% to 36% of patients in published OHVIRA cohorts, attributed to retrograde flow from the obstructed side [6]. In a retrospective cohort study applying the Enzian classification across the spectrum of Müllerian anomalies, a rudimentary horn containing a functional cavity carried a markedly higher prevalence of endometriosis than anomalies without functional tissue - of the order of two-thirds versus one-quarter of patients [7]. Neither estimate comes from a cohort restricted to unicornuate uterus with a rudimentary horn, so the comparison with the present case is illustrative rather than directly applicable, though both point toward a strong association between a functional obstructed segment and endometriosis. The coexistence of deep infiltrating endometriosis extending to the pelvic sidewall, an ovarian endometrioma, and diffuse adenomyosis is consistent with, and may have contributed to, retrograde menstruation from a functioning rudimentary horn and the patient's chronic pelvic pain, while her dyschezia is more specifically attributable to the deep infiltrating nodule tenting the rectum; given the multiple coexisting lesions, these symptom-lesion associations are clinically plausible rather than established with certainty [6,7]. Mapping deep infiltrating disease, including the distance of the nodule from the anal margin and the absence of transmural rectal invasion, feeds directly into surgical planning and is part of what the imaging contributes beyond the classification itself.

What makes this case worth reporting is the simultaneous occurrence of four findings that are individually described but rarely documented together in one patient: a unicornuate uterus with a rudimentary horn, an adenomyoma within that horn, deep infiltrating endometriosis, and ipsilateral renal agenesis. Each pairwise association appears in the literature - Müllerian anomaly with renal agenesis, unicornuate uterus with endometriosis, and adenomyoma mimicking a malformation - but their convergence, and the way MRI resolved a long-standing misclassification while mapping every component in a single study, is uncommon and is the principal contribution here. Individual reports have paired a non-communicating rudimentary horn with ipsilateral renal agenesis and either adenomyosis or endometriosis [11,12], or with a solitary pelvic kidney and endometriosis presenting atypically as an inguinal hernia [13], but none combine a rudimentary horn adenomyoma, deep infiltrating endometriosis, and ipsilateral renal agenesis in the same patient.

The principal limitation of this report is its single-case design, which precludes conclusions regarding prevalence, causality, or the broader applicability of the imaging findings. In addition, although the MRI diagnosis was confirmed surgically and histopathologically, the imaging features described may not represent the full spectrum of unicornuate uterus with a rudimentary horn or its associated complications. Communication of the rudimentary horn was assessed only indirectly, from the presence of hematometra without dilation of the functional cavity on MRI; hysterosalpingography, hysteroscopy, and intraoperative probing were not performed to confirm this directly. Nevertheless, the case offers complete radiologic-pathologic correlation and demonstrates the pivotal role of MRI in correcting a long-standing misclassification, identifying associated renal anomalies, and accurately defining the extent of coexisting deep infiltrating endometriosis for surgical planning.

## Conclusions

A uterus recorded as didelphys was shown on MRI to be a unicornuate uterus with a rudimentary horn containing an adenomyoma, a distinction that had gone uncorrected for years. Accurate classification informed surgical planning in this patient and is clinically relevant more broadly, because a functional rudimentary horn carries risks - including hematometra, endometriosis, and rudimentary horn pregnancy - that a diagnosis of didelphys would not have flagged. The associated left renal agenesis is consistent with the shared embryologic relationship between the genital and urinary tracts and should be sought routinely, and the same examination characterized the coexistent deep infiltrating endometriosis and ovarian endometrioma; this coexistence provides a plausible explanation for the patient's symptoms and is consistent with a possible contribution from retrograde menstruation. Standard sagittal, axial, and coronal planes were sufficient to establish the diagnosis in this patient; where a Müllerian anomaly is suspected, however, aligning the coronal T2-weighted sequence to the uterine long axis is a recognized technique that may further improve assessment of uterine morphology, although these findings, drawn from a single case, should not be generalized without support from larger studies.
